# Comment on 'Blinatumomab vs historical standard therapy of adult relapsed/refractory acute lymphoblastic leukemia'

**DOI:** 10.1038/bcj.2016.119

**Published:** 2016-12-09

**Authors:** W H Tong

**Affiliations:** 1 Department of Hospital Medicine, Haaglanden Medical Center (HMC), The Hague, The Netherlands; 2 Department of Internal Medicine, Haaglanden Medical Center (HMC), The Hague, The Netherlands

I have read the contribution of Gökbuget *et al.*^1^ with interest. The authors compared outcomes of a single-arm study of blinatumomab in adult patients with B-precursor Ph-negative relapsed/refractory acute lymphoblastic leukemia (R/R ALL) with a historical dataset from Europe and the United States. The clinical efficacy of blinatumomab against the historical data was analyzed with different statistical methods and sensitivity. However, some important statistical questions can be raised, which I address below.

First, was a pre-planned statistical analysis plan designed prior to the data collection? This is mandatory according to the guidelines of the International Conference on Harmonisation of Technical Requirements for Registration of Pharmaceuticals for Human Use (ICH) and the principles of Good Clinical Practice (GCP).^2^ Additionally, this study was not a placebo-controlled trial. It would be of interest to show the stratified and weighted analysis results when excluding the patients of the era 1990–1999 as the number of patients with salvage treatment in that era is rather small compared with the era 2000 onwards. Also, in this and a previous paper from these authors, it was shown that the complete remission and overall survival values differed significantly between the periods 1990–1994, 1995–1999, 2000–2004 and 2005 onwards.^3^

Second, Amgen as a pharmaceutical company was responsible for the statistical analysis. Was there any substantial ground to have their department of Biostatistics to apply these rather difficult statistical techniques? In general, the used techniques are not easy to understand for the general hematologist.

Third, possibly unmeasured confounders could have a role in a comparison with a historical dataset. This should be accounted for. In the Supplementary Table 2, the participant data are clearly shown by country. However, it is not clear whether the group created by combining the patients from France, Italy and the United States was sufficiently homogeneous for the final analysis. And why were not raw survival data presented and separated by the most important prognostic variables in comparison with the overall survival in the blinatumomab clinical trial?

Fourth, from a methodological point of view: why were the outlier values truncated? The measured parameters are designated as results. If an outlier was found, the statistical model predicted this value as an outlier. Presumably, this statistical model is not completely correct.

Lastly, the use of propensity scores in this study may not be appropriate. The authors should have corrected for *a priori* relevant variables according to the ICH and GCP guidelines. Therefore, it is suggested not to construct a weighted co-variable afterwards.

To conclude, the authors used two analytical approaches in evaluating the efficacy of blinatumomab versus current treatments by using the largest available dataset of adult patients with Ph-negative B-precursor R/R ALL. The appropriateness of these approaches can be questioned. To avoid any potential influencing and bias of a pharmaceutical company, it is recommended to apply difficult statistical approaches by an independent academic department of Biostatistics without any conflict of interest.
